# Nonspecific Feelings Expected and Experienced during or Immediately after Electroacupuncture: A Pilot Study in a Teaching Situation

**DOI:** 10.3390/medicines4020019

**Published:** 2017-04-08

**Authors:** David F. Mayor, Lara S. McClure, J. Helgi Clayton McClure

**Affiliations:** 1 Department of Allied Health Professions and Midwifery, School of Health and Social Work, University of Hertfordshire, Hatfield AL10 9AB, UK; 2 Northern College of Acupuncture, York YO1 6LJ, UK; LaraMcClure@chinese-medicine.co.uk (L.S.M.); helgi.claytonmcclure@oxon.org (J.H.C.M.)

**Keywords:** electroacupuncture, nonspecific feelings, expectation, placebo, *qi*, cluster analysis

## Abstract

**Background:** Some feelings elicited by acupuncture-type interventions are “nonspecific”, interpretable as resulting from the placebo effect, our own self-healing capacities—or, indeed, the flow of *qi*. Expectation is thought to contribute to these nonspecific effects. Here we describe the use of two innovative 20-item questionnaires (EXPre_20_ and EXPost_20_) in a teaching situation. **Methods:** Respondents were acupuncture students or practitioners on electroacupuncture (EA) training courses (*N* = 68). EXPre_20_ and EXPost_20_ questionnaires were completed before and after receiving individualised treatment administered by colleagues. Respondents were also asked about their prior experience of EA or transcutaneous electroacupuncture stimulation (TEAS). **Results:** Respondents *expected* significantly more items to change than not to change, but significantly fewer were *experienced* as changing. Increases in given questionnaire items were both expected and experienced significantly more often than decreases. “Tingling”, “Relaxation”, and “Relief” or “Warmth” were most often expected to increase or were experienced as such, and “Pain” and “Tension” to decrease or experienced as decreasing. Expectations of change or no change were confirmed more often than not, particularly for “Tingling” and “Tension”. This was not the result of the personal respondent style. Cluster analysis suggested the existence of two primary feeling clusters, “Relaxation” and “Alertness”. **Conclusions:** Feelings experienced during or immediately after acupuncture-type interventions may depend both on prior experience and expectation.

## 1. Introduction

Writings about acupuncture often mention its “nonspecific” effects, although even those familiar with the literature on these effects vary in their interpretation of the term [1]. The placebo effect is sometimes considered as evidence for an activation of what some consider as our “nonspecific” self-healing capacities [2,3,4]. There are also accounts of how, in response to placebo (more accurately, sham) acupuncture, bodily sensations of warmth, tingling, pulsing, flow (spreading, radiating), and electricity have been elicited—warmth and tingling being particularly associated with treatment efficacy [5,6]. Such sensations are also reported in other complementary and alternative medicine (CAM) modalities such as “biofield energy healing” [7], and have been interpreted by many CAM practitioners as resulting from the flow of *qi*, the immanent life force of the body and the world (part agency, part image or form, part metaphor), of key importance in acupuncture and Chinese culture as a whole, as well as being central to Western traditions of vitalism, where it has many other names [8,9].

The expectation of a positive outcome is thought to be a major contributor to nonspecific effects of treatment [10], partly because it alters how bodily sensations are identified [11]. However, no independently developed pre-existing questionnaires were found that could be used to assess expectation or experience of the nonspecific feelings that may arise in response to acupuncture-type interventions, although scales have been developed to evaluate the common specific sensations elicited by acupuncture [12,13,14,15,16,17,18,19,20,21]. Our objectives in this study were therefore to use two previously developed 20-item questionnaires, EXPre_20_ and EXPost_20_, to investigate differences in response patterns between respondent subgroups and identify any patterns of change (both expected and experienced) for particular nonspecific feelings. For further explanation on these “EXP_20_” questionnaires and their precursors, EXPre_32_ and EPost_32_, see Section 2, Materials and Methods, below, and for the questionnaires themselves, see Appendix A and Appendix B).

**Research questions and hypotheses**

1. Firstly, the study aims to address the question of fulfillment of expectation. Our central hypothesis is that there will be strong, generally positive correlations between the expected and experienced feelings—in other words, that expectation in this context is generally fulfilled [10,11]. (This should not be conflated with the association between expectation of treatment *effectiveness* and its outcome, or benefit [22,23,24]).

2. We aim to establish whether there are trends across responses to different questionnaire items. For instance, there might be significant overall differences between the numbers of “Yes” and “No” responses, or between the numbers of “increase” and “decrease” responses, given to the various nonspecific feelings assessed by the questionnaires. For example, feelings of “Relaxation” have been reported in response to acupuncture treatment [25], and feelings of “Aliveness” might be associated on theoretical grounds with an improvement in the flow of *qi* [8]. Would both these be found to increase in this context?

3. We also aim to address the question of prior experience effects. Those with prior experience of electroacupuncture (EA) or transcutaneous electrical nerve stimulation (TENS) might report generally different expectations or experiences of feelings elicited by EA/TEAS (transcutaneous electroacupuncture stimulation) than are reported by those without. Related to this, students might report different expectations or experiences than those reported by practitioners. Our use of Continuing Professional Development (CPD) practitioner versus Student respondent groups, as well as recording the presence versus absence of prior experience of EA/TENS, will allow us to explore the effects of the treatment experience in two different ways (general and specific).

Of course, differences by respondent group or by prior experience might be detected *between* questionnaire items also.

4. A final key aim of the present study is to identify any significant associations among the different feelings assessed by the questionnaires. A cluster analysis will enable us to better understand these associations, through an exploration of the relationships between them. For example, using the language of Chinese medicine, some feelings could be considered more “*yang*” (masculine, positive, expansive) and others more “*yin*” (feminine, negative, withdrawing) [26]. Are these ideational associations reflected in the clusters found?

## 2. Materials and Methods

### 2.1. Recruitment, Questionnaires, and Treatments

Respondents were recruited during pre-arranged electroacupuncture (EA) teaching sessions in the UK, three of these being university-affiliated undergraduate acupuncture training courses and two independently organised CPD courses for acupuncture practitioners (in Nottingham and Brighton). The basic structure of the sessions was similar on all courses, although one of the undergraduate courses (CICM) was only a half-day rather than a full-day session. The same lecturer—an acupuncturist with over 30 years of experience—presented and supervised all the courses.

**Figure 1:**
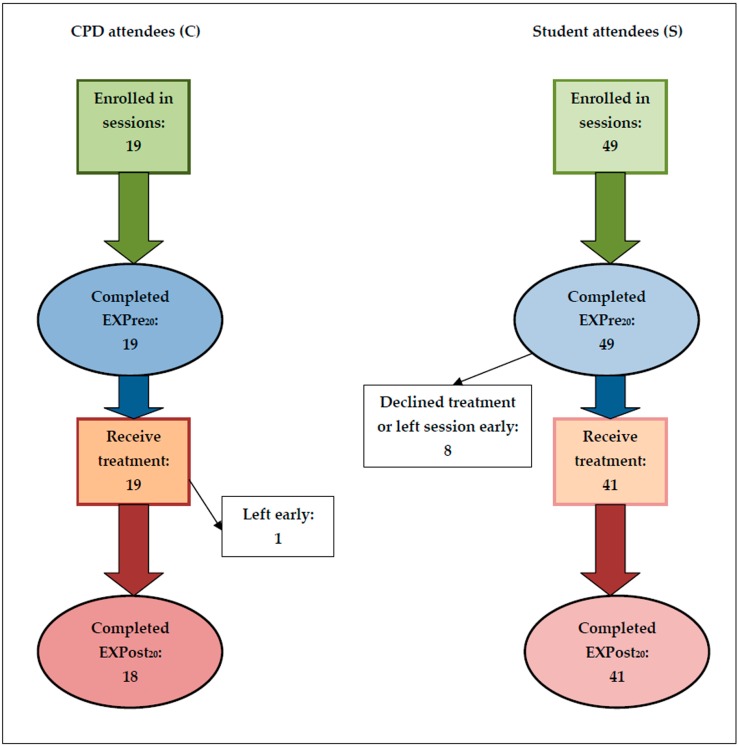
Study flow chart for CPD (C) and Student (S) attendees. Key: EXPre_20_ = initial expectation questionnaire; EXPost_20_ = follow-up experience questionnaire.

Respondents were asked to complete two 20-item questionnaires: an initial expectation questionnaire (EXPre_20_) at the beginning of the teaching session, and a follow-up experience questionnaire (EXPost_20_) after receiving a short treatment from a fellow attendee (a flow chart of this sequence is shown in Figure 1).

**Pilot Studies: development of the EXP_20_ questionnaires from earlier 32-item versions**

In earlier research, two longer 32-item questionnaires had been piloted in three cohorts of acupuncture and other complementary health practitioners and students familiar with acupuncture, who received EA or TEAS (transcutaneous electroacupuncture stimulation as against the usual “nerve stimulation”, TENS) in experimental studies or a classroom situation (*N* = 204). They were designed to assess expectation (EXPre_32_) and experience (EXPost_32_) of the relatively nonspecific feelings (bodily, emotional, or mental) that may arise in response to acupuncture-type interventions, in particular the established methods of EA and TEAS [27]. Findings on their content validity and reliability, together with a cluster analysis, have been presented elsewhere [28]. The salient results were that significant numbers of feelings experienced by respondents were those they expected, and that significant numbers of feelings not experienced were those not expected. It should be noted that many of the participants in this earlier study had no prior experience of EA/TEAS, although nearly all were familiar with acupuncture.

Following this, 20 experienced acupuncture practitioners and researchers rated items in the original 32-item questionnaires as either “essential”, “useful but not essential”, or “not necessary”. These ratings, together with the analysis of actual questionnaire usage, were used to reduce the original questionnaires from 32 to 20 items [29]. More information on the development of the EXP_20_ questionnaires can be found in the publications cited [28,29,30].

Treatment was supervised but participants were free in this teaching situation to use their own choice of acupuncture points and stimulation parameters (frequency, amplitude, mode, pulse, and overall stimulation duration). They were also encouraged to use several different EA/TEAS stimulators, of which 11 were provided for their use. Treatments were carried out in small groups of 3–4 participants. Information on the treatments received is provided in the Supplementary Material Table S1.

### 2.2. Ethics Approval

Ethics approval was granted under an application for a related study by the Health and Human Sciences Ethics Committee of the University of Hertfordshire, UK (Protocol HEPEC/07/11/93), approved 5 July 2011, 24 Aug 2012 . Permission was also received from course organisers and respondents.

### 2.3. Questionnaire Administration

The present paper describes and analyses the use of the shorter 20-item questionnaires in a teaching situation (*N* = 68).

The questionnaires (Appendix B) were printed with items in randomised order, so it was unlikely that they would appear in the same order in both EXPre_20_ and EXPost_20_ for a particular respondent. These two were also distributed and collected separately, so that they could not be seen at the same time, reducing the likelihood of respondents basing their replies to EXPost_20_ on their earlier replies to EXPre_20_.

In EXPre_20_, respondents were asked “Relative to how you feel NOW, during or immediately following EA/TEAS do you expect to experience any change AT ALL in the feeling of …” (a particular feeling). Possible responses were “Yes”, “No”, or “don’t know/can’t say” (“DK”, i.e., no particular expectation). If they answered “Yes”, they were then asked if they expected the feeling to increase or decrease. Similarly, in EXPost_20_, respondents were asked “Relative to how you felt when you completed the earlier questionnaire, during or immediately following EA/TEAS did you experience a change in the feeling of …”.

Questionnaires were collated using pre-printed ID codes double-checked against respondents’ signatures/initials and writing styles.

### 2.4. Statistical Analysis

Anonymised scores for each questionnaire item were analysed using Excel and SPSS v20. Binomial and χ^2^ (chi-square) tests were used to assess significance of differences; to assess the degree of association, Cramer’s V and Pearson’s r were used (both with a range of 0 to 1). Three methods of hierarchical cluster analysis appropriate for binary data were used: Jaccard’s index (or “similarity ratio”), Sokal and Sneath’s index 5 (range 0 to 1) [31,32], and Ward’s method. For the first two methods, distances were calculated using average linkage (both between and within groups), nearest neighbour (single linkage), and furthest neighbour (complete linkage). Squared Euclidean distances were used for Ward’s method. Appropriate numbers of clusters were estimated visually from the dendrograms for each method, particularly where there was apparent agreement among them for the largest reasonable (readily interpretable) number of clusters [32].

## 3. Results

### 3.1. Respondents

The present study sample consisted of acupuncture practitioners undertaking continuous professional development courses (hereafter CPD or practitioners) and students of acupuncture (Students). The two groups were composed of respondents from five training centres (CPD: Brighton, Nottingham; Students: the College of Integrated Chinese Medicine, Reading (CICM), London South bank University (LSBU), and the Northern College of Acupuncture, York (NCA)).

Student respondents were obliged to attend EA sessions as part of their acupuncture training, whereas practitioner respondents signed up for EA courses voluntarily (all respondents were informed that they did not need to complete the questionnaires if they did not wish to). Training centre, age, gender (where known), prior experience of EA and/or TENS, and numbers completing the two questionnaires are shown in Table 1. All attendees completed the initial questionnaire. A few attendees declined treatment because of known contraindications (e.g., pregnancy or a heart condition), an aversion to “non-traditional” EA/TEAS, or to electricity itself. Some students left the sessions early, and thus were not present to complete EXPost_20_ questionnaires. The flow chart in Figure 1 shows the numbers at the start of the EA sessions and those at the end.

As would be expected, students were consistently younger than practitioners (*p* < 0.001 for the difference in their ages, using an independent-samples *t*-test, with *t*(64) = 3.8). As for the gender of the attendees, even if all missing cases were men, there is still a significant preponderance of women (*p* = 0.001 using the Binomial test with a test proportion of 0.5). Again, for CAM practitioners, this would be expected [33].

Details of the treatments received are summarised in Supplementary Materials Table S1.

### 3.2. Research Question 1: Fulfilment of Expectation

#### 3.2.1. Overall Patterns of Expectation

**Figure 2:**
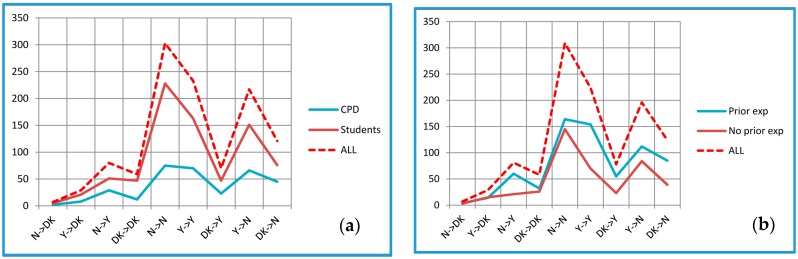
Relationships between counts of expected and experienced “Yes” (Y), “No” (N), and “Don’t know” (DK) change scores: (**a**) CPD and student subgroups; (**b**) Subgroups of those with prior or no prior experience of EA/TENS.

Although individuals’ responses varied considerably, overall there were clear patterns of the relationships between the counts of expected and experienced “Y”, “N”, and “DK” change scores, shown in Figure 2 as “N→DK”, etc.

Thus the expectations of change, whether negative or positive, were confirmed (N→N and Y→Y) more often than other combinations, the next most common combination being expected changes that were not confirmed in practice. DK→N also outnumbered DK→Y. (Similar patterns were found for the earlier versions of the questionnaires, EXPre_32_ and EXPost_32_ [28]). Furthermore, in each cohort except for the Brighton CPD group, significantly more respondents showed confirmatory scores than would be expected by chance (*p* ≤ 0.001, using the Binomial test with a test proportion of 0.25, i.e., based on N→N and Y→Y, but ignoring the DK→DK responses).

When all items were considered together, there were positive linear correlations between items expected to change and experienced as changing (r = 0.869), and also between those not expected to change and not experienced as changing (r = 0.722). There were negative correlations between items not expected to change and those experienced as changing (r = 0.727) and vice versa (r = 0.832).

Only in one cohort (Brighton, *N* = 11) were expectations less often confirmed than not (for Y→N, but not N→Y).

Comparing the results for CPD and students, only for DK→N was the difference between these two subgroups significant (*p* = 0.018).

Those with prior experience of EA/TENS showed rather more N→Y and Y→Y scores than those without prior experience (*p* < 0.001). Differences between these two subgroups were also significant for DK→Y (*p* = 0.002) and DK→N (*p* = 0.001).

For increases (i) and decreases (d), there were no significant differences between when expectations of change were confirmed (i→i or d→d) or not (i→d or d→i) (*p* > 0.05). However, counts of each of these four combinations were higher for those with prior experience of EA/TENS than for those without prior experience, as indicated in Supplementary Materials Figure S1 (see also p 9 below).

When all the items were considered together, there were positive linear correlations between items expected to increase and experienced as increasing (r = 0.750), and also between those expected to decrease and experienced as decreasing (r = 0.916). These correlations were driven by increases in Relaxation, Tingling and Warmth, and by decreases in Being stressed, Pain and Tension. If these were removed from the analysis, the apparent linearity was no longer evident (r = 0.446 and r = 0.173, respectively).

There was no indication that respondents who tended to answer one way to the EXPre_20_ questionnaire (as “increasers”, “i”, or “decreasers”, “d” [29]) were likely to answer the same way to the EXPost_20_ questionnaire (*p* > 0.05).

#### 3.2.2. Patterns of Expectation for Individual Questionnaire Items

Counts of the various change responses for individual items were made. The highest counts for the various EXPre_20_/EXPOst_20_ combinations are shown in Supplementary Materials Table S3. For DK→DK and DK→N, two items were tied in first position, and for Y→DK, three items.

Items above the third quartile for Y→Y were Tingling (count 40), Relaxation (20), Pain and Tension (16), and Warmth (15), all of which were included in EXP_32_ (although not among the Y→Y items above the third quartile there). The third quartile N→N items in EXP_20_ were Cheerfulness (26), Clarity and Heaviness (23), Sleepiness (22), and Being spaced out (21). Again, although included in EXP_32_, they did not occur in the third quartile EXP_32_ N→N items. The case for DK→DK is similar.

Expected/experienced increases and decreases are shown in Supplementary Materials Table S4.

The results for Tingling and Tension are in line with those in the previous table. Those for Relaxation (inc→dec) and Pain (dec→inc) are somewhat surprising (but involve only small numbers).

### 3.3. Research Question 2: Individual Questionnaire Items—Expectations of Change, Increase and Decrease

#### 3.3.1. Changes/No Changes and Increases/Decreases Most and Least Expected

Questionnaire item counts were ranked and the results were tabulated. Those above the third quartile (75th percentile, in the “top five”) are shown in Table 2.

#### 3.3.2. Correlations between Items Expected to Change/Not Change, or Increase/Decrease

There is evidently some correspondence between items most expected to change and those least expected not to change (three items in common) and vice versa (four items in common). Taking all items into account, there was a strong negative linear correlation between those expected to change and those not expected to change (r = −0.893).

There is less correspondence between those items most expected to increase and those least expected to decrease (two items in common), and more between those items most expected to decrease and those least expected to increase (four items in common). Compared with expectations of change, there was a relatively small negative correlation between items expected to increase and those expected to decrease (r = −0.510).

### 3.4. Research Question 2: Individual Questionnaire Items—Experiences of Change, Increase and Decrease

#### 3.4.1. Changes/No Changes and Increases/Decreases Most and Least Experienced

Questionnaire item counts were ranked and the results were tabulated. Those above the third quartile (75th percentile, in the “top five”) are shown in Table 3. In addition, respondents were asked explicitly to asterisk changes they “noticed most” (see Appendix B-2). Only 16 did so (12 students, 4 practitioners), with 42 items asterisked between them (1–8 items per respondent, mode 2). The numbers of asterisked items are included (in parentheses) in Table 3. Other items asterisked but not above the third quartiles (not included in Table 3) were Aliveness (1), Being spaced out (2), Calmness (3), Heaviness (2), Inner bodily flow (1), Mental energy (1), and Sleepiness (4). Wellbeing was the only item not asterisked.

Here there is a similar degree of agreement between greater experience of change and lesser experience of no change (four items in common), and between greater experience of no change and lesser experience of change (four items in common). Taking all item counts into consideration, there was a negative linear correlation between those experienced as changing and not changing (r = −0.944). This was stronger than the correlation for the expected items.

Apart from Calmness and Sleepiness (asterisked three and four times), and Relief (asterisked twice), there is agreement between those items for which changes were most *often* experienced and those experienced with most *intensity* (“noticed most”, and asterisked three times or more).

#### 3.4.2. Correlations between Items Most/Least Experienced as Increasing/Decreasing

There is some correspondence between those items most experienced as increasing and those least experienced as decreasing (three items in common), but less between those items most experienced as decreasing and those least experienced as increasing (two items in common). There was a very weak negative correlation between items experienced as increasing and those experienced as decreasing (r = −0.358).

#### 3.4.3. Ratios of “Yes”/”No” and “Increase”/”Decrease” Score Counts

Significance of the ratios of “Yes”/”No” and “increase”/”decrease” score counts are shown in Supplementary Materials Table S2, together with the sign of the difference between the counts.

Only for “Cheerfulness” was there a significant Expected/Not expected change ratio in both the EXPre_20_ and EXPost_20_. In contrast, 12 items (60%) showed a significant increased/decreased ratio in both EXPre_20_ and EXPost_20_. For all these 12 items, increases outnumbered decreases (whether expected or experienced).

### 3.5. Research Question —Differences in Response Patterns between Respondent Subgroups

#### 3.5.1. Drop-outs, i.e., Those Not Completing the EXPost20 Questionnaire

Nine respondents did not complete the EXPost_20_ questionnaire. Eight of these (more than expected) had no prior experience of EA/TENS, and were also students (*p* = 0.039 each, using the ratio test).

#### 3.5.2. Those with and without Prior Experience of EA or TENS

Overall, similar numbers had (36) and had not (31) had prior experience of EA/TENS, with proportionally more in the practitioner/CPD cohorts having prior experience (however, this was a nonsignificant difference).

#### 3.5.3. Prior Experience and Expectation

For no single EXPre_20_ item was the Binomial test for those expecting a change significant. “Tingling” was the only item significant (*p* = 0.025) for those not expecting a change (none of those without prior experience expected no change in this item). Those who did expect a change in “Tingling” were divided almost equally between those with prior experience (28) and those without (27). For all EXPre_20_ items taken together, however, the expectation of change and the expectation of increase approached significance for the 0.54 test proportion (and would have been significant had group sizes been equal).

Significantly more of those with prior experience were uncertain whether an increase or decrease was expected (*p* = 0.023; *p* = 0.001 for test proportion 0.50).

#### 3.5.4. Practitioner and Student Expectation

No significant differences in expectation of change/no change were found between students and practitioners. Practitioners expected fewer decreases than students (*p* = 0.024), and were less likely to report uncertainty in their expectation of increase or decrease (*p* = 0.003). No differences were significant for any individual item.

#### 3.5.5. Practitioner and Student Questionnaire Responses

Across all questionnaire items in EXPre_20_, practitioners recorded 142 “yes” responses (106 increases, 26 decreases), 111 “no” responses, and 78 no-expectation responses. Students recorded 419 “yes” responses (294 increases, 104 decreases), 314 “no” responses, and 187 no-expectation responses.

Across all questionnaire items in EXPost_20_, practitioners recorded 126 “yes” responses (72 increases, 17 decreases), 189 “no” responses, and 22 no-expectation responses. Students recorded 264 “yes” responses (202 increases, 50 decreases), 458 “no” responses, and 74 no-expectation responses.

Unspecified “yes” responses in both cases were those where neither increase nor decrease was indicated.

Across all responses in EXPre_20_ there was no significant difference in the distribution between practitioners and students (*p* > 0.05). Similarly, there was no significant difference with respect to the direction of change (“increase” or “decrease”). Across all responses in EXPost_20_, again the distributions were not significantly different when missing data responses were excluded (*p* > 0.05). In subsequent analyses (other than in Section 3.5 below), “don’t know” (DK) and missing data responses were disregarded.

#### 3.5.6. Ratios of “Yes” and “No” Counts in Questionnaire Responses

Ratios of “Yes”/”No” and “increase”/”decrease” counts are shown in Table 4 and Table 5, respectively.

All the EXPre_20_ count ratios, except for the practitioner EXPre_20_ “Yes/No” ratio and the practitioner EXPost_20_/EXPre_20_ “Yes” ratio, are significantly different from 1 (*p* < 0.001).

Obvious patterns are also that “Yes” responses outnumber “No’s” in EXPre_20_, but “No” responses outnumber “Yes” responses in EXPost_20_, and that “No” responses in EXPost_20_ outnumber those in EXPre_20_, but that “Yes” responses in EXPre_20_ outnumber those in EXPost_20_. (Similar results were found for EXPre_32_ and EXPost_32_ [28]).

Again, all count ratios are significantly different from 1 (*p* < 0.001) except for the practitioner EXPost_20_/EXPre_20_ “increase” ratio (*p* = 0.001) and the practitioner “decrease” ratio (n.s.).

However, whereas the ratios of the change counts (Table 4) are quite dissimilar (median 1.76, interquartile range [IQR] 0.67–4.05), the “increase”/”decrease” ratios (Table 5) are quite similar for both EXPre_20_ and EXPost_20_, as are the EXPost_20_/EXPre_20_ ratios for both “increase” and “decrease” (median 1.09, IQR 0.66%–1.36%).

### 3.6. Research Question 4—Associations between Different Items and Exploratory Cluster Analysis

#### 3.6.1. Associations between Pairs of Items

**Figure 3:**
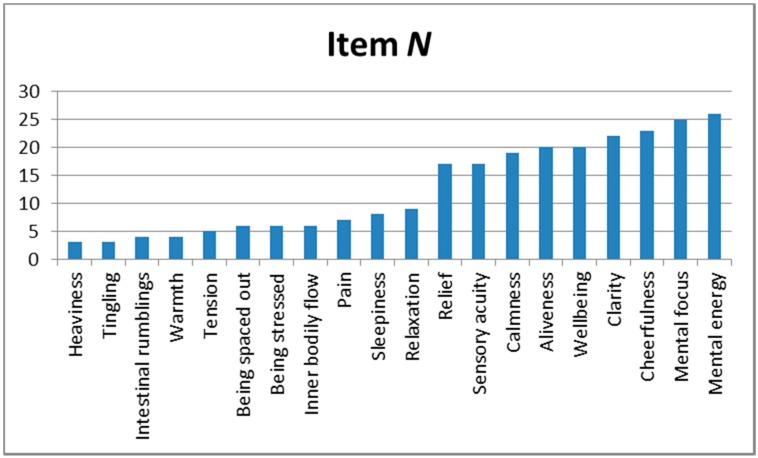
Number of times each item appeared in item pairs with a medium or high level of Cramer’s V.

Cramer’s V was used as a simple method of assessing how closely the different items were associated, based on the categorical scores (“Y” or “N”) allocated by the respondents. Low values of V (<0.3) were ignored (V ≥0.3 is considered by Cohen to indicate a medium level of association, and V ≥0.5 a high level [34]). Figure 3 shows how frequently each item appeared in item pairs with a medium or high level of Cramer’s V.

Using Cramer’s V, there was a higher percentage (with a higher average V) of items showing significant EXPre_20_-EXPre_20_ associations than of items with significant EXPre_20_-EXPost_20_ or EXPost_20_-EXPost_20_ associations.

There are two subgroups of items here: a lower one (mean occurrence rate 5.5, range 3–9) and an upper one (mean occurrence rate 21.0, range 17–26). Of the EXPre_20_-EXPre_20_ pairs, 24 of 35 (68.6%) consisted of items only in the upper subgroup, of the EXPost_20_-EXPost_20_ pairs, 28 of 46 (60.9%), and of the EXPre_20_-EXPost_20_ pairs, 31 of 46 (67.4%).

#### 3.6.2. Cluster Analysis

Numbers of estimated clusters using Jaccard’s index, Sokal and Sneath’s index 5, and Ward’s method are shown in Supplementary Materials Table S5. Items in the clusters obtained using the different methods were compared, and those for which there was the most agreement were selected.

A comparison between Cramer’s V and Ward’s proximities showed no obvious relationship between the two measures overall. However, in both EXPre_20_ and EXPost_20_ two clusters stood out from all the others, having the highest mean V and lowest mean proximity. These could be considered as clusters for “Relaxation” and “Alertness”. The values of mean Ward proximities (W) and Cramer’s V for the two clusters suggest that “Alertness” was the more robust of the two (Supplementary Materials Table S6). Other possible clusters were “Relief” and “Bodily sensation”, but there was less agreement between EXPre_20_ and EXPost_20_ on the items included.

If the data was split by subgroup (CPD vs. student respondents, or with vs. without prior experience of EA/TENS), no combinations of items appeared in corresponding clusters for all four subgroups. In the EXPre_20_ responses, the Calmness/Relaxation dyad did not appear in any cluster for those with no prior experience of EA/TENS, and in the EXPost_20_ responses only in the student subgroup. In the EXPre_20_ responses, the triad of Aliveness, Cheerfulness, and Mental focus appeared together in a cluster for all subgroups except for that of students, and that of Cheerfulness, Mental energy, and Mental focus in a cluster for all but the subgroup with prior experience of EA/TENS. In the EXPost_20_ responses, the dyad of Aliveness and Mental energy and the tetrad of Cheerfulness, Clarity, Mental focus, and Sensory acuity appeared together (albeit in separate clusters) for all subgroups except for those with no prior experience of EA/TENS. Subgroup analysis was not carried out for the EXPre_20_ and EXPost_20_ items taken together.

## 4. Discussion

### 4.1. Respondents

The pattern evident in those respondents who only filled out the EXPre_20_ and not the EXPost_20_ questionnaire suggests that those who did not complete the second questionnaire (predominantly students) had not really become more interested in EA/TEAS after the course than they were before attending. This could reflect a failure of teaching skill on the part of the instructor, or a lack of openness to something outside the normal (“traditional”) curriculum among students, for whom this was an obligatory session (whereas the CPD respondents signed up for their sessions because of an interest in what was being taught). In addition, some of the students will have left the session early to ensure they were able to catch their usual transport home.

Practitioners tended to have somewhat more prior experience of the EA and TENS modalities than students. Given that the students were all enrolled in “traditional” acupuncture training courses, this was to be expected, even if the difference between the practitioners and students did not reach significance in this respect.

Somewhat surprisingly, six of the 36 respondents who had prior experience of EA/TENS expected no change in “Tingling”, whereas none of the 31 without such prior experience expected no change in “Tingling”. Electricity is commonly associated with a “Tingling” feeling [8].

Those with no prior experience of EA/TENS showed less uncertainty in their expectations of increases or decreases in feelings than those who did have prior experience. Real life clinical experience may soften the certainty of preconceptions.

### 4.2. Questionnaire Items—Overall Patterns

Overall, students and practitioners scored the questionnaires in a similar way. In particular, whereas more respondents expected feelings to change than did not expect them to, fewer respondents actually experienced changes in feelings (cf [28]). Thus there were fewer EXPost_20_ than EXPre_20_ “Yes” responses, but more “No” responses.

In contrast, more “increases” than “decreases” were both expected and experienced (with a slightly higher ratio of “increases” to “decreases” in EXPost_20_ than EXPre_20_). As there were fewer “Yes” counts following treatment than before, the EXPost_20_/EXPre_20_ ratios for both “increases” and “decreases” were all <1.

### 4.3. Individual Questionnaire Items—Expectations of Change, Increase and Decrease

Some responses might be self-evident to anyone familiar with any complementary therapy: following a treatment, a change (increase) in relaxation or relief would be expected or hoped for, and also a change (decrease) in pain or tension.

Inner bodily flow (which might be expected on the basis of prior experience of or teachings on energy-based medicine [8], and could be interpreted by some respondents in terms of electrical current flow) was considered less likely to change or increase by those with prior experience. Heaviness and Sleepiness were both among those items considered least likely to change AND those items likely to decrease. Calmness and Heaviness were considered likely to change by CPD respondents, but less so by the students.

### 4.4. Individual Questionnaire Items—Experiences of Change, Increase and Decrease

The changes most commonly experienced (Pain, Relaxation, Tension, Tingling, Warmth) were similar to those expected (Pain, Relaxation, Relief, Tension, Tingling), with Relaxation, Tingling, and Warmth among the items most often increasing, and Pain and Tension among those most often decreasing. There was overall agreement between those items for which changes were most *often* experienced and those experienced with most *intensity* (“noticed most”).

Sleepiness was among the items most experienced as not changing for the (CPD/no prior experience) respondents, Heaviness for the (student/no prior experience) respondents, and Calmness and Mental focus for all those with no prior experience. Conversely, Relief was among the items most experienced as changing for the CPD and no prior experience respondents. The no prior experience subgroup therefore appears to have had a different experience of what did and did not change than the others.

Of the 12 items showing significantly more increases than decreases in both EXPre_20_ and EXPost_20_, all could be considered as “positive” in the sense of increasing with overall wellbeing rather than decreasing.

### 4.5. Fulfilment of Expectation

A key finding is that—as for the EXP_32_ questionnaires—expectations of change, whether negative or positive, were confirmed rather than not. Only in one cohort (*N* = 11) was this not the case.

Positive expectations of change were more marked among those with prior experience of EA/TENS than those without.

In contrast, expectations of increase or decrease were not fulfilled (rather than confirmed) by experience. However, there were significantly more counts of all four combinations of EXPre_20_ and EXPost_20_ “i” and “d” scores from respondents with prior experience of EA/TENS than from those without. Further study would be required to confirm these findings, as missing data rates were high (31 of 561, or 5.5%, for EXPre_20_, and 49 of 390, or 12.6%, for EXPost_20_).

That 10 out of the 59 (17%) respondents who completed both the EXPre_20_ and EXPost_20_ questionnaires reported a change in Being stressed, whereas they had expected not to, is concordant with the experienced decreases shown in Table 3 above (where “Being stressed” was included among the items above the third quartile). Eleven (19%) who were uncertain if they would experience a change in the feeling of Being stressed experienced no change, and 18 (31%) who expected a change in the feeling of tension experienced no change.

Nine of those who were uncertain if they would experience a change in warmth in fact did (it being one of the items most experienced as increasing, as shown in Table 3), whereas 11 (19%) who were uncertain if they would experience a change in Intestinal rumblings also did not (this, like Sensory acuity and Mental focus, being an item that was least experienced as either increasing or decreasing, as shown in Table 3).

Taking Table 2, Table 3 and Tables S2–S4 together, the salient items are Relaxation, Tingling and Warmth, and Pain and Tension. Future research into the feelings elicited by EA and TEAS should at least take these into account (Cheerfulness also appears frequently in the Tables, but mostly because little change in this feeling was expected or experienced).

Associations were more evident between EXPre_20_-EXPre_20_ item pairs than between EXPre_20_-EXPost_20_ or EXPost_20_-EXPost_20_ pairs. Counts of items occurring in pairs with medium or high levels of Cramer’s V showed that they fell into two groups, one of relatively low counts (range 3–9), and one with higher counts (range 17–26). Cluster analysis suggested the existence of two clusters for both EXPre_20_ and EXPost_20_ items, one which could be considered as indicating “Relaxation”, and the other “Alertness”. The latter appeared more robust; the two clusters considered together are redolent of the traditional acupuncture concepts of *yin* and *yang* [26].

Although there was no obvious relationship between Cramer’s V and Ward’s proximities, all but one of the items in the “Alertness” cluster were all in the higher count range for Cramer’s V. In contrast, of the two items consistently occurring in the “Relaxation” cluster, the “Relaxation” item itself was in the lower count range (albeit at the top end of that range).

Further research using the EXP_20_ questionnaires should be conducted to replicate our findings and explore their application in different contexts, in particular in more rigorously designed clinical studies, and in relation to mainstream or CAM treatments other than EA. They could also be applied outside academic institutions, and even in everyday life situations. Such research should take into account the various issues flagged under “Limitations”, described below.

## 5. Conclusions

Our main findings were that expectations of change, whether negative or positive, were confirmed rather than not, and that the changes most commonly experienced (Pain, Relaxation, Tension, Tingling, Warmth) were indeed similar to those expected (Pain, Relaxation, Relief, Tension, Tingling), with Relaxation, Tingling, and Warmth among the items most often increasing, and Pain and Tension among those most often decreasing. Cluster analysis suggested the existence of two primary clusters for both EXPre_20_ and EXPost_20_ items, one which could be considered as indicating “Relaxation” (consisting of the items Calmness and Relaxation), the other “Alertness” (Aliveness, Cheerfulness, [Clarity], Mental energy, Mental focus, and Sensory acuity).

It is hoped that the EXP_20_ questionnaires will be used by other researchers to replicate these findings, and also be developed further. It would be interesting, for example, to see whether results differ for men and women, and also whether different feelings are elicited by different types of acupuncture (in particular, sham acupuncture where significant debate exists surrounding the assumed inertia of the intervention [21,35,36]). They could perhaps also be used with outcome measures to explore whether “good responders” tend to experience complementary therapy treatments in a way that is different from those who respond less well.

### 5.1. Limitations

Attendees were not asked to provide information about their gender. Where available, this data was gathered retrospectively for each cohort, so that it is not possible to relate individuals’ responses and their gender. Given the preponderance of women in the study (at least 68% and possibly as high as 81%), it is highly likely that our results are valid for women alone, but further research will be required to confirm that findings are valid for men as well as for women.

CPD attendees were not asked how many years they had been in practice. Differences between them could have impacted both the treatments they gave and their expectations and experiences of treatment effects. It would require a larger study to explore this factor.

CPD respondents attended these EA teaching sessions voluntarily, whereas the students did not. This may have had an impact on how seriously they took the task of completing the questionnaires. Nonetheless, this does not appear to have led to major differences between the C and S respondents (other than for Research question 1).

Because this study was conducted in teaching situations where attendees from different acupuncture training backgrounds were encouraged to explore the techniques of EA and TEAS for themselves, the treatments given were very heterogeneous. Beyond suggesting that it was good practice to obtain a *deqi* response before applying electrical stimulation through the needles, no attempt was made to control the needling technique or to change the methods of needling with which the attendees were already comfortable.

Furthermore, this is a small pilot study on participants familiar with acupuncture and the subjective sensations it may elicit. It is not known how far the results can be extrapolated to the wider population who are likely to be less familiar with such sensations, nor how they would be reflected in a purely clinical context.

A potential weakness in the test procedure concerns the contamination of responses to the later (ExPost_20_) questionnaire. Small group discussion on the EA/TEAS techniques used was encouraged during the treatment exchange sessions before this questionnaire was administered, so that even though individuals completed it independently, their responses may have been somewhat influenced by others’ comments. However, it is important to note that this effect is likely to be minimal since time was limited, the treatment/discussion groups were indeed small (*N* = 3 or 4), and the focus of the discussion was on the *technicalities* of EA/TEAS rather than on participants’ subjective experience. Furthermore, although the “grain size” of the resulting ExPost_20_ data may have been fairly coarse, it is highly unlikely that there was contamination *between* the small groups. In our view, despite the strong contrary opinion of our most rigorous anonymous reviewer, the results still support our conclusions regarding fulfillment of expectation, since: (A) the initial (ExPre_20_) questionnaire was completed with no potential for contamination; (B) ExPost_20_ was presented with a separately randomised question order, without recourse to ExPre_20_ responses; (C) although there may conceivably have been contamination of responses within some of the small groups, it is highly unlikely that this was so consistent as to explain our findings; and (D) results were similar across the different cohorts. In other words, the experiences reported by the respondents were consistently in accord with and very likely influenced by their expectations (and not just their earlier reporting of expectations), and any within-group distortion was minimal.

Of course it must be kept in mind that the data analysed in the present study represent inherently subjective reports of feelings and experiences. There are therefore likely to be many factors contributing to the responses both to ExPre_20_ and ExPost_20_, the fine-grain investigation of which was not within the scope of this present study.

Finally, whereas most respondents were able to score most items for expected or experienced changes, there were more lacunae in the data for increases/decreases. Because of this missing data, the results for expected/experienced increases and decreases are less certain, and should be confirmed in further studies with more respondents.

## Figures and Tables

**Table 1:** Respondent cohorts, showing age (mean, SD), gender, whether practitioner/CPD (C) or student (S), prior experience of electroacupuncture (EA) and/or transcutaneous electrical nerve stimulation (TENS), and numbers completing each of the two questionnaires, EXPre_20_ and EXPost_20_, as well as both EXP questionnaires.

Training Centre	Total *N*	Age	Gender	C/S	Prior	No Prior	EXPre_20_	EXPost_20_
LSBU (19.10.13)	14	37.8, 8.3	11 F, 2 M	S	8	6	14	11
Nottingham (23.11.13)	8	52.1, 5.7	n/a ^a^	C	6	2	8	7
Brighton (06.04.14)	11	45.9, 7.9	9 F, 2 M	C	6 ^b^	4 ^b^	11	11
CICM (07.04.14)	24	39.7, 9.5	19 F, 5 M	S	10	14	24	19
NCA (12.04.14)	11	39.8, 9.8	9 F, 2 M	S	6	5	11	11
*CPD N*	*19*	*48.3*, *7.6*	*9–17 F*, *2–10 M *^a^	*C*	*12*	*6*	*19*	*18*
*Student N*	*49*	*39.1*, *9.1*	*39 F*, *9 M*	*S*	*24*	*25*	*49*	*41*
Total *N*	68	41.7, 9.6	48–56 F, 11–19 M	n/a	36	31	68	59

^a^ Information not available; ^b^ One respondent did not answer this question.

**Table 2:** Changes/no changes and increases/decreases most and least expected. Items listed are for the whole sample. Subgroups in which the listed items did not occur are shown in the “not sub” columns.

Change Most *Expected*	Not Sub	No Change Least *Expected*	Not Sub	Change Least *Expected*	Not Sub	No Change Most *Expected*	Not Sub
Tingling *	-	Tingling	-	Cheerfulness	-	Cheerfulness	-
Relaxation *	n	Warmth	s	Being spaced out	-	Being spaced out	-
Tension *	c	Inner bodily flow	c	Sensory acuity	-	Heaviness	c
Pain	-	Relief	n	Sleepiness	s, n	Sleepiness	n
Relief	c, n	Relaxation	-	Clarity	c, n	Clarity	-
**Increase Most *Expected***	**Not Sub**	**Decrease Least *Expected***	**Not Sub**	**Increase Least *Expected***	**Not Sub**	**Decrease Most *Expected***	**Not Sub**
**Tingling**	-	Cheerfulness	-	Being stressed	-	**Pain**	-
**Relaxation**	-	Aliveness	-	Pain	-	**Tension**	-
Warmth	c	Wellbeing	-	Cheerfulness	-	Being stressed	-
**Relief**	c, n	Warmth	-	Tension	n	Heaviness	-
Wellbeing	s, n	(5 tied items ^†^)	-	Heaviness	c, p	Sleepiness	c

Key: c = CPD; s = students; p = prior experience of EA/TENS; n = no prior experience of EA/TENS. Items in bold are those for which the most agreement occurred for “change” as well as for either “increase” or “decrease”. * Agreement with results for EXPre_32_ [27]; ^†^ Clarity, Inner bodily flow, Mental energy, Mental focus, Sensory acuity.

**Table 3:** Changes/no changes and increases/decreases most and least experienced. Items listed are for the whole sample. Subgroups in which the listed items did not occur are shown in the “not sub” columns.

Change Most *Experienced*	Not Sub	No change Least *Experienced*	Not Sub	Change Least *Experienced*	Not Sub	No change Most *Experienced*	Not Sub
Tingling (3)	-	Tingling (3)	-	Mental focus (2)	n	Intestinal rumblings * (2)	-
Relaxation * (6)	-	Pain (4)	p	Intestinal rumblings (2)	-	Sensory acuity (0)	-
Warmth (4)	-	Relief (2)	-	Sensory acuity (0)	-	Being stressed (0)	s,n
Pain (4)	s,p	Relaxation (6)	-	Cheerfulness (1)	-	Clarity (1)	-
Tension * (3)	c	Warmth (4)	n	Clarity (1)	-	Mental focus (2)	c,n
**Increase Most *Experienced***	**Not Sub**	**Decrease Least *Experienced***	**Not sub**	**Increase Least *Experienced***	**Not Sub**	**Decrease Most *Experienced***	**Not Sub**
**Tingling**	-	Sensory acuity	-	Mental focus	-	**Tension**	-
**Relaxation**	-	Inner bodily flow	-	Being stressed	-	**Pain**	-
**Warmth**	-	Warmth	-	Tension	n	Being stressed	-
Being spaced out	c,n	Mental focus	n	Sensory acuity	p	Aliveness	-
Calmness	c,n	(6 tied items ^†^)	-	Intestinal rumblings	n	Heaviness	n

Key: c = CPD; s = students; p = prior experience of EA/TENS; n = no prior experience of EA/TENS. Numbers in square brackets indicate the numbers of an item asterisked as “most noticed” by respondents. Items in bold are those for which the most agreement occurred for “change” as well as for either “increase” or “decrease”. * Agreement with results for EXPre_32_ [27]; ^†^ Being spaced out, Calmness, Cheerfulness, Clarity, Intestinal rumblings, Tingling.

**Table 4:** Ratios of “Yes” and “No” counts for EXP_20_.

EXP_20_	Y/N (Pre)	Y/N (Post)	Y (Post/Pre)	N (Post/Pre)
*CPD*	1.28 ** (n.s.)	0.67 **	0.89 (n.s.)	1.70 **
*Student*	1.33 **	0.58 **	0.63 **	1.46 **
Total	1.32 **	0.60 **	0.70 **	1.52 **

** *p* < 0.001 (Binomial test, test ratio 0.50).

**Table 5:** Ratios of “increase” and “decrease” counts for EXP_20_.

Subgroup	Inc/Dec (Pre)	Inc/Dec (Post)	Inc (Post/Pre)	Dec (Post/Pre)
*CPD*	4.07 **	4.24 **	0.68 *	0.65 (n.s.)
*Student*	2.83 **	4.04 **	0.69 **	0.48 **
Total	3.08 **	4.09 **	0.69 **	0.52 **

* *p* = 0.001; ** *p *< 0.001

## Supplementary Materials

The following are available online at www.mdpi.com/2305-6320/4/2/19/s1, Table S1: Respondent cohorts, Table S2: Differences between score counts (change/no change, increase/decrease) for the EXPre_20_ and EXPost_20_ items, Table S3: Questionnaire items with the highest counts for the various “expected”/”experienced” combinations, Table S4: Questionnaire items with the highest counts for the various “increase” and “decrease” combinations, Table S5: Numbers of estimated clusters of EXPre_20_, EXPost_20_ and EXPre_20_ *and* EXPost_20_ items; Figure S1: Relationships between counts of expected and experienced increase (i) and decrease (d) scores.
