# Supplementary material for: Nonspecific Feelings Expected and Experienced during or Immediately after Electroacupuncture: A Pilot Study in a Teaching Situation
Source: Medicines (Basel). 2017 Apr 8;4(2):19. doi: 10.3390/medicines4020019 (PMC5590055; doi:10.3390/medicines4020019)
Supplement: Supplementary file 1 [file medicines-04-00019-s001.docx]

Supplementary Materials: Nonspecific feelings expected and experienced during or immediately after electroacupuncture: A pilot study in a teaching situation

David F. Mayor, Lara S. McClure and J. Helgi Clayton McClure

**Table S1.** Summary of treatments received, showing the number of points used in each bodily region, the number of points per treatment (pts/Rx), treatment frequencies used (Hz), and treatment durations (in minutes).

| **Points used** | ***N*** | ***N* pts/Rx** |  | **Hz** | ***N*** | **Duration** | ***N*** |
| --- | --- | --- | --- | --- | --- | --- | --- |
| Face/head | 7 | 2 | 47^a^ | Low | 28 | < 5 min | 4 |
| Neck/shoulder | 37 | 4 | 8 | 10-20 Hz | 14 | 5 min | 28 |
| Upper limb | 41 | 6 | 2 | 25-35 Hz | 5 | 10 min | 24 |
| Back | 9 |  |  | High | 24 | 15 min | 14 |
| Lower limb | 34 |  |  | DD^b^ | >3 | 20 min | 5 |
| Other *ashi* | 8^a^ |  |  | Various | 5 |  |  |

a. *Ashi* points in undisclosed bodily regions were mentioned by four respondents; these have been entered as paired; b. Several respondents mentioned that they had used ‘various’ or ‘high and low’ frequencies, others that they used ‘dense-disperse’ stimulation (i.e. alternating high and low frequencies); it is not clear to what extent these descriptions overlap.

**Table S2.** Differences between score counts (change/no change, increase/decrease) for the EXPre_20_ and EXPost_20_ items. Significance of the difference is shown, together with the sign of the difference. Where this sign changed between EXPre_20_ and EXPost_20_, it is bracketed.

| **ALL** | **EXPre_20_** | | | | **EXPost_20_** | | | |
| --- | --- | --- | --- | --- | --- | --- | --- | --- |
| (Binomial  p values) | Change – no change |  | Increase – decrease |  | Change – no change |  | Increase – decrease |  |
| Aliveness | n.s. | + | <0.001 | + | 0.040 | [-] | n.s. | [-] |
| Being spaced out | 0.016 | - | n.s. | + | n.s. | - | <0.001 | + |
| Being stressed | n.s. | - | 0.002 | - | 0.001 | - | n.s. | - |
| Calmness | n.s. | + | **<0.001** | + | n.s. | [-] | **<0.001** | + |
| Cheerfulness | **<0.001** | - | **0.004** | + | **0.001** | - | **0.021** | + |
| Clarity | n.s. | - | **0.001** | + | <0.001 | - | **0.021** | + |
| Heaviness | n.s. | - | n.s. | - | 0.013 | - | n.s. | [+] |
| Inner bodily flow | 0.010 | + | **<0.001** | + | n.s. | [-] | **<0.001** | + |
| Intestinal rumblings | n.s. | = | **0.004** | + | <0.001 | [-] | **0.039** | + |
| Mental energy | n.s. | + | **<0.001** | + | 0.005 | [-] | **0.013** | + |
| Mental focus | n.s. | = | <0.001 | + | <0.001 | [-] | n.s. | + |
| Pain | 0.016 | + | <0.001 | - | n.s. | + | n.s. | - |
| Relaxation | 0.001 | + | **<0.001** | + | n.s. | + | **<0.001** | + |
| Relief | 0.001 | + | **<0.001** | + | n.s. | [-] | **0.001** | + |
| Sensory acuity | n.s. | - | **0.001** | + | <0.001 | - | **0.008** | + |
| Sleepiness | n.s. | - | n.s. | + | 0.030 | - | 0.001 | + |
| Tension | 0.002 | + | 0.003 | - | n.s. | [-] | n.s. | - |
| Tingling | <0.001 | + | **<0.001** | + | <0.001 | [-] | **<0.001** | + |
| Warmth | 0.001 | + | **<0.001** | + | n.s. | + | **<0.001** | + |
| Wellbeing | n.s. | + | **<0.001** | + | n.s. | [-] | **0.007** | + |
| significant/sign | 9 items (7 +) |  | 17 items (14 +) |  | 11 items (all -) |  | 14 (all +) |  |

**Table S3.** Questionnaire items with the highest counts for the various ‘expected’/’experienced’ combinations, showing the count (*N*) for each item, the number of counts for that item for all changes (Y🡪Y, Y🡪N, etc.), and their relative percentage. Underlined items are those found for the same combination in EXP_32_; asterisked items were not included in EXP_32_.

| **EXPre🡪EXPost** | **Agreement for complete sample** | ***N*** | ***N* ALL changes** | **%** |
| --- | --- | --- | --- | --- |
| N🡪DK | Cheerfulness | 2 | 54 | 4% |
| Y🡪DK | Calmness, Pain, Wellbeing* | 4 | 58, 55, 56 | 7% |
| N🡪Y | Being stressed* | 10 | 57 | 18% |
| DK🡪DK | Inner bodily flow, Mental focus | 6 | 55 | 11% |
| N🡪N | Cheerfulness | 26 | 54 | 48% |
| Y🡪Y | Tingling | 40 | 56 | 71% |
| DK🡪Y | Warmth | 9 | 57 | 16% |
| Y🡪N | Tension | 18 | 56 | 32% |
| DK🡪N | Being stressed*, Intestinal rumblings | 11 | 57 | 19% |

**Table S4.** Questionnaire items with the highest counts for the various ‘increase’ and ’decrease’ combinations, showing the count (*N*) for each item, the number of counts for that item for all changes (Y🡪Y, Y🡪N, etc.), and their relative percentage.

| **EXPre🡪EXPost** | **Agreement for complete sample** | ***N*** | ***N* ALL changes** | **%** |
| --- | --- | --- | --- | --- |
| inc🡪inc | Tingling | 28 | 56 | 50% |
| inc🡪dec | Pain, Relaxation, Tension | 2 | 55,55,56 | 4% |
| dec🡪dec | Tension | 12 | 56 | 21% |
| dec🡪inc | Pain, Sleepiness | 3 | 55 | 5% |

**Table S5.** Numbers of estimated clusters of EXPre_20_, EXPost_20_ and EXPre_20_ *and* EXPost_20_ items using Jaccard’s index, Sokal and Sneath’s index 5 (S & S 5) and Ward’s method.

| **Clustering method** | **EXPre_20_-EXPre_20_** | **EXPost_20_-EXPost_20_** | **EXPre_20_-EXPost_20_** |
| --- | --- | --- | --- |
| Jaccard (between) | 5 | 4 | 8 |
| Jaccard (within) | 6 | 7 | 8 |
| Jaccard (nearest n) | 7 | 7 | 8 |
| Jaccard (furthest n) | 6 | 7 | 8 |
| S & S 5 (between) | 7 | 7 | 8 |
| S & S 5 (within) | 5 | 7 | 7 |
| S & S 5 (nearest n) | 7 | 8 | 9 |
| S & S 5 (furthest n) | 6 | 7 | 9 |
| Ward (Sq Euclidean) | 6 | 6 | 8 |
| Mode | 6 | 7 | 8 |

**Table S6.** ‘Relaxation’ and ‘Alertness’ clusters in the EXPre_20_, EXPost_20_ and EXPre_20_ *and* EXPost_20_ data, with mean Ward proximities (W) and Cramer’s V (V) for each cluster (omitting items in parentheses).

| **Cluster** | **EXPre_20_** | **EXPost_20_** | **W; V** | **EXPre_20_ and EXPost_20_** | **W; V** |
| --- | --- | --- | --- | --- | --- |
| ‘Relaxation’ | (Being stressed)^a^  Calmness  Relaxation  (Wellbeing)^d^ | Calmness  Relaxation | 7.0; 0.6 | Calmness (pre)  Relaxation (pre)  Calmness (post)^d^  Relaxation (post)^d^ | 1.0; 0.5 |
| ‘Alertness’ | Aliveness  Cheerfulness  Mental energy  Mental focus  (Sensory acuity)^b^ | Aliveness  Cheerfulness  Clarity  Mental energy  Mental focus  Sensory acuity | 3.7; 0.5 | Cheerfulness (pre)  Mental focus (pre)  Clarity (post)  Mental focus (post)  Sensory acuity (post)  Wellbeing (post)^d^  (Mental energy (pre))^c^  (Aliveness (post))^b^  (Heaviness (post))^b^  (Mental energy (post))^b^ | 0.0; 0.5 |

a. Agreement for 8 out of the 9 methods used; b. Agreement for 6 of the 9 methods; c. Agreement for 5 of the 9 methods; d. Agreement for 4 of the 9 methods.


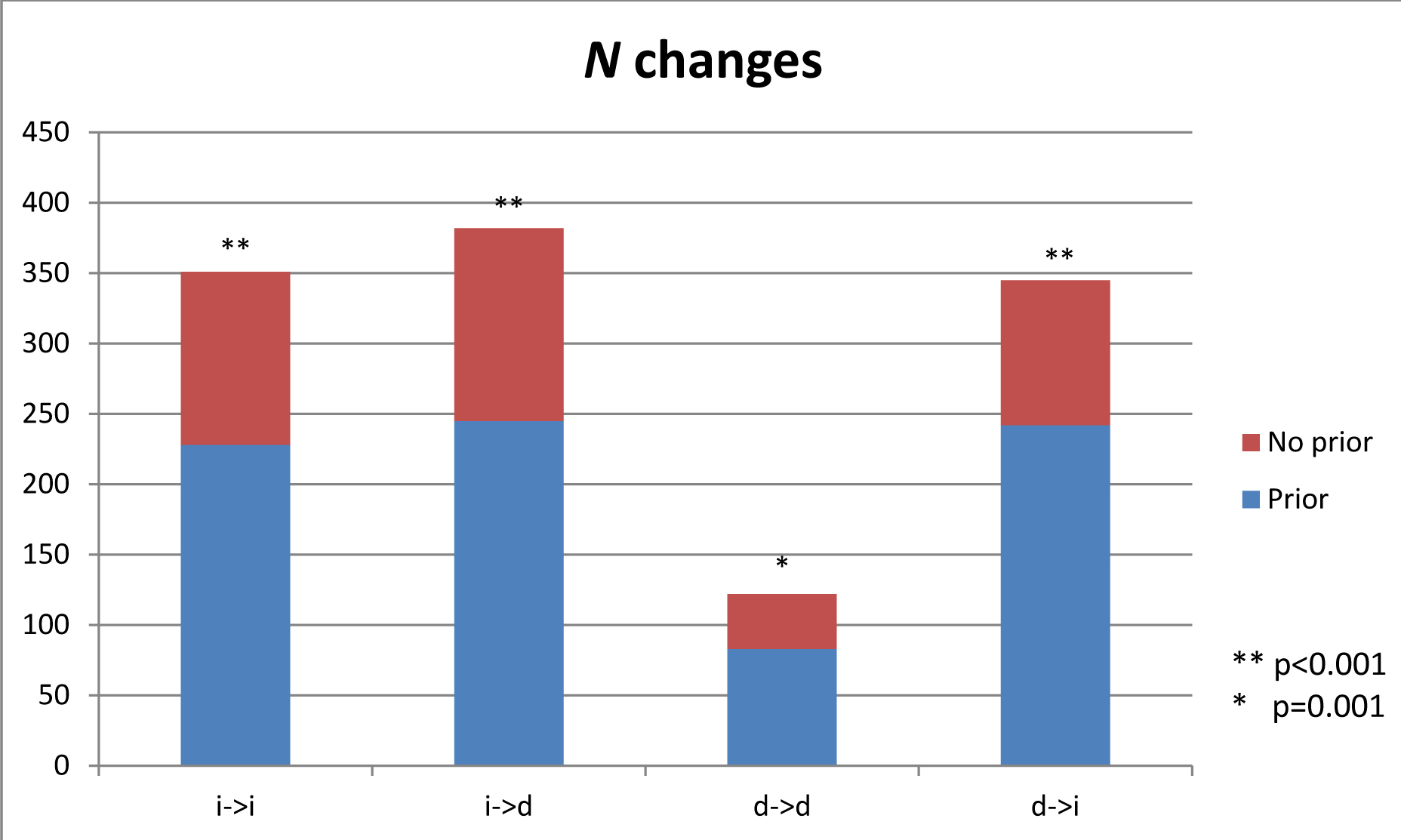


**Figure S1.** Relationships between counts of expected and experienced increase (i) and decrease (d) scores, showing significance of differences between respondents with and without prior experience of EA/TENS.
